# PNPLA3 I148M Polymorphism, Clinical Presentation, and Survival in Patients with Hepatocellular Carcinoma

**DOI:** 10.1371/journal.pone.0075982

**Published:** 2013-10-14

**Authors:** Luca Valenti, Benedetta Maria Motta, Giorgio Soardo, Massimo Iavarone, Benedetta Donati, Angelo Sangiovanni, Alessia Carnelutti, Paola Dongiovanni, Raffaela Rametta, Cristina Bertelli, Floriana Facchetti, Massimo Colombo, Silvia Fargion, Anna Ludovica Fracanzani

**Affiliations:** 1 Department of Pathophysiology and Transplantation, Università degli Studi di Milano, Milano, Italy; 2 Internal Medicine, Fondazione Istituto di Ricovero e Cura a Carattere Scientifico (IRCCS) Ca' Granda - Ospedale Maggiore Policlinico Milano, Milano, Italy; 3 Internal Medicine, Università di Udine, Udine, Italy; 4 First Diviosion of Gastroenterology, Fondazione Istituto di Ricovero e Cura a Carattere Scientifico (IRCCS) Ca' Granda - Ospedale Maggiore Policlinico Milano, Milano, Italy; Bambino Gesu' Children Hospital, Italy

## Abstract

**Background & Aims:**

Aim of this study was to evaluate whether the PNPLA3 I148M polymorphism, previously associated with hepatocellular carcinoma (HCC) risk, influences the clinical presentation of HCC and survival.

**Methods:**

we considered 460 consecutive HCC patients referred to tertiary care centers in Northern Italy, 353 with follow-up data.

**Results:**

Homozygosity for PNPLA3 148M at risk allele was enriched in HCC patients with alcoholic liver disease or nonalcoholic fatty liver disease (ALD&NAFLD: relative risk 5.9, 95% c.i. 3.5–9.9; other liver diseases: relative risk 1.9, 95% c.i. 1.1–3.4). In ALD&NAFLD patients, the PNPLA3 148M allele was associated with younger age, shorter history of cirrhosis, less advanced (Child A) cirrhosis at HCC diagnosis, and lower HCC differentiation grade (p<0.05). Homozygosity for PNPLA3 148M was associated with reduced survival in the overall series (p = 0.009), and with a higher number of HCC lesions at presentation (p = 0.007) and reduced survival in ALD&NAFLD patients (p = 0.003; median survival 30, 95% c.i. 20–39 vs. 45, 95% c.i. 38–52 months), but not in those with HCC related to other etiologies (p = 0.86; 48, 95% c.i. 32–64 vs. 55, 95% c.i. 43–67 months). At multivariate Cox regression analysis, homozygosity for PNPLA3 148M was the only negative predictor of survival in ALD&NAFLD patients (HR of death 1.57, 95% c.i. 1.12–2.78).

**Conclusions:**

PNPLA3 148M is over-represented in ALD&NAFLD HCC patients, and is associated with occurrence at a less advanced stage of liver disease in ALD&NAFLD. In ALD&NAFLD, PNPLA3 148M is associated with more diffuse HCC at presentation, and with reduced survival.

## Introduction

Hepatocellular carcinoma (HCC) is most frequently related to cirrhosis resulting from chronic liver diseases caused by hepatitis B and C viruses, alcohol, obesity, and rare genetic disorders. Environmental toxins, diabetes, and family history represent common predisposing factors [1], but genetic variants have also been demonstrated to modify HCC susceptibility [2].

In particular, the rs738409 C>G polymorphism, encoding for the functional I148M protein variant in the patatin-like phospholipase domain-containing 3 (PNPLA3, adiponutrin) gene has been associated with the risk of developing HCC [3], [4], [5], [6], [7], [8], [9], [10]. The I148M PNPLA3 polymorphism is the major genetic determinant of hepatic fat content and liver enzymes in the general population [11], [12], [13]. Furthermore, the 148M risk allele favors disease progression in nonalcoholic fatty liver disease (NAFLD) [14], [15], alcoholic liver disease (ALD) [16], [17], chronic hepatitis C (HCV) [4], [18], and also in liver diseases unrelated to steatosis [19], [20]. Interestingly, the I148M polymorphism affects HCC risk independently of the effect on fibrosis [4], [5], [6], [7], [10], [21], suggesting that it has a direct carcinogenic effect by modulating lipid metabolism or inflammatory mediators [3], [5], [6], [7], [8], [9]. Furthermore, incorporation of the I148M polymorphism in model scores improved HCC risk stratification in patients with alcoholic cirrhosis [8], thereby representing an attractive biomarker for individualization of the clinical management of ALD.

However, the implications of PNPLA3 genotype for patients already diagnoses with HCC are still unknown, as only preliminary and controversial data are available on the influence of I148M polymorphism on the clinical presentation and outcome of HCC [9], [22].

We hypothesized that PNPLA3 I148M predisposes to a specific carcinogenic pathway associated with dysregulation of hepatic lipid metabolism [23], [24], [25], resulting in HCC with distinct biological features. Therefore, aim of this study was to evaluate whether the I148M polymorphism is associated with the clinical presentation and survival in a series of HCC patients from Northern Italy.

## Materials and Methods

### Patients

In this retrospective-prospective study, we considered 460 patients with HCC diagnosed at, or referred to, tertiary referral centers in Northern Italy, for whom DNA samples and clinical data were available. Most of the patients were diagnosed during regular screening for chronic liver disease (369/460, 80%). The case series included 254 consecutive patients evaluated or treated at the Internal Medicine service, University of Milan, between January 2000 and June 2012, 120 consecutive patients treated at the Internal Medicine service, University of Udine between January 2011 and June 2012, and 86 patients treated at the Gastroenterology service, University of Milan, between January 2010 and June 2012. HCC was diagnosed according to the European and American scientific associations guidelines [26], [27], [28]. Before 2001 we considered only patients with histological diagnosis of HCC. Fifty/460 (11%) of the described patients were included in a previous study from our group [4].

For each patient we recorded the age at diagnosis, gender, presence / absence of cirrhosis, year of diagnosis of cirrhosis and of HCC, Child-Pugh classification of cirrhosis, presence of diabetes, daily alcohol intake. The etiology of liver disease was classified as related to HCV, chronic HBV infection (HBV), HBV + HCV, ALD, NAFLD, hereditary hemochromatosis (HH), or cryptogenic; based on clinical history, daily alcohol intake, physical examination, and assessment of viral markers (including HBsAg, anti-HBc antibodies, and HCV-RNA) in all patients. This series did not include patients with liver disease related to autoimmune liver disease, Wilson disease, or alpha1-anti-trypsin deficiency. In the presence of viral infection, patients were classified as HBV or HCV even if cofactors (such as obesity, at risk alcohol intake, or autoimmunity) were present. Patients were classified as affected by cryptogenic liver disease if viral markers were negative, and no iron overload or history of obesity, at risk alcohol intake, or known genetic diseases were present (even if burn-out NASH could not be excluded). The body mass index was not considered because of the confounding effect of cachexia and fluid retention in patients with advanced disease.

The number of HCC lesions at diagnosis, the size of the single or of the major HCC lesion at diagnosis, Barcelona Clinic Liver Cancer (BCLC) HCC stage at diagnosis (0, A–B, or C–D), prospective follow-up with recording of time of death or liver transplantation was available in 356 patients (77%). Evaluation of Edmonson histological grade of HCC at diagnosis was available in 118 patients [29].

In the case of remission, follow-up was carried up at 3–6 months or shorter intervals according to the clinical requirements of the patients. No patient was lost at follow-up. The time of death was confirmed by telephone calls with the relatives of the patients. Data were censored at the time of death or liver transplantation (15 patients were transplanted) or July 1^st^, 2012.

Patients were treated according to the European and American scientific associations guidelines [26], [27], [28], by multidisciplinary teams (one common team in Milan and one in Udine) encompassing medical clinicians, surgeons (at liver transplant centers), and interventional radiologists. Although the treatment algorithm changed during the 12 years of the study, in particular after the introduction of sorafenib in clinical practice for patients with advanced disease, all centers had access to the full panel of treatment options, and clinicians were unaware of the genetic background of the patients, rendering the presence of a bias unlikely.

As a reference group for the frequency distribution of the I148M polymorphism in healthy patients without liver disease (which did not allow to evaluate the effect of the I148M polymorphism on HCC incidence), we considered 257 healthy blood donors with normal liver enzymes (ALT <35/30 IU/ml in M/F, GGT <35 IU/ml) and metabolic parameters, with a very low probability of steatosis [30].

Informed written consent was obtained from each subject. The study conforms to the ethical guidelines of the 1975 declaration of Helsinki and was approved by the Institutional Review Board of the Fondazione Ca' Granda and participating centers.

Demographic and clinical features of subjects included are presented in Table 1. The clinical features of patients with complete clinical data and follow-up were not significantly different from those of the other patients (Table S1).

**Table 1:** Demographic, clinical, and genetic features of subjects included in the study.

	HCC	Reference group
n =	460	257
Age years	66±9	48±12
Sex F	97 (21)	55 (21)
Diabetes	118 (26)	0
HCV	279 (61)	0
HBV	65 (14)	0
Alcohol abuse	80 (17)	0
Cirrhosis	442 (96)	0
PNPLA3 I148M		
I/I	194 (42)	146 (57)
I/M	187 (41)	95 (37)
M/M	79 (17)	16 (6)

(): % values; HCC: hepatocellular carcinoma; HCV: chronic hepatitis C virus infection, HBV: chronic hepatitis B virus infection (co-infected patients were considered in both categories), F: female, PNPLA3: patatin-like phosholipase domain-containing 3. p = 1.8×10^−6^ for the frequency distribution of the I148M polymorphism between HCC patients and controls.

### Genetic analysis

The rs738409 C>G (I148M) PNPLA3 polymorphism was genotyped in a blinded fashion on DNA specimens obtained by peripheral blood by a 5′nuclease Taqman assay (assay on demand for rs738409, Applied Biosystems, Foster City, CA), as previously described [14] at the Metabolic Liver Disease Laboratory at Fondazione IRCCS Ca' Granda. DNA was successfully extracted and genotyped from each subject included in the study. Internal controls were used in each batch analyzed.

### Statistical analysis

Results are expressed as means ± standard deviation or median {interquartile range} according to data distribution. Mean values were compared by Anova, and frequencies by Chi-square test, according to data distribution, and differences were considered significant when p≤0.05 (two-tailed). Non-normally distributed variables were log-transformed before analysis. Based on the previous literature, a recessive model was used to assess the association of the I148M polymorphism with clinical presentation of the patients and survival [25]. An additive model was also tested for the association with clinical features of HCC patients at diagnosis. The association between the PNPLA3 I148M polymorphism with HCC was evaluated by multivariate logistic regression analysis adjusted for age and gender. The association between the PNPLA3 I148M polymorphism and survival (recessive model) was evaluated by Kaplan-Meier at univariate analysis (Log-rank test), and Cox regression models adjusted for confounding variables, which included those selected *a priori* for their biological relevance, plus those which were found associated with the outcome of interest at univariate analysis (specified in the Results section). P values for model fitness were <0.05. Our sample size had > 95% power of detecting an association between PNPLA3 I148M and survival with an OR of 2.0 with a significance of 5%. Analyses were carried out with the statistical analysis software JMP 9.0 (SAS Institute Inc, Cary, NC), and SPSS 19 (IBM, Armonk, NY).

## Results

### Frequency of I148M PNPLA3 polymorphism in patients and healthy subjects

The frequency distribution of the I148M polymorphism was significantly different between HCC patients and the reference group (shown in Table 1; p = 1.8×10^−6^), due to over-representation of homozygosity for the 148M allele (Table 1; p = 1.4×10^−5^) previously associated with steatosis. The association was also evident in the 356 patients with complete data and follow-up (Table S1; p = 9.4×10^−6^ for the frequency distribution, p = 4.4×10^−6^ for the prevalence of 148M homozygosity).

### I148M PNPLA3 polymorphism and etiology of HCC

Demographic, clinical, and genetic features of HCC patients according to the etiology of liver disease are shown in Table S2. Age at diagnosis, gender, and the severity of liver disease were significantly different according to etiology. Interestingly, HCC arose more frequently in patients without cirrhosis in patients with NAFLD than in those with other etiologies (4/28, 14% vs. 13/432, 3%; p = 0.015).

**Figure 1:**
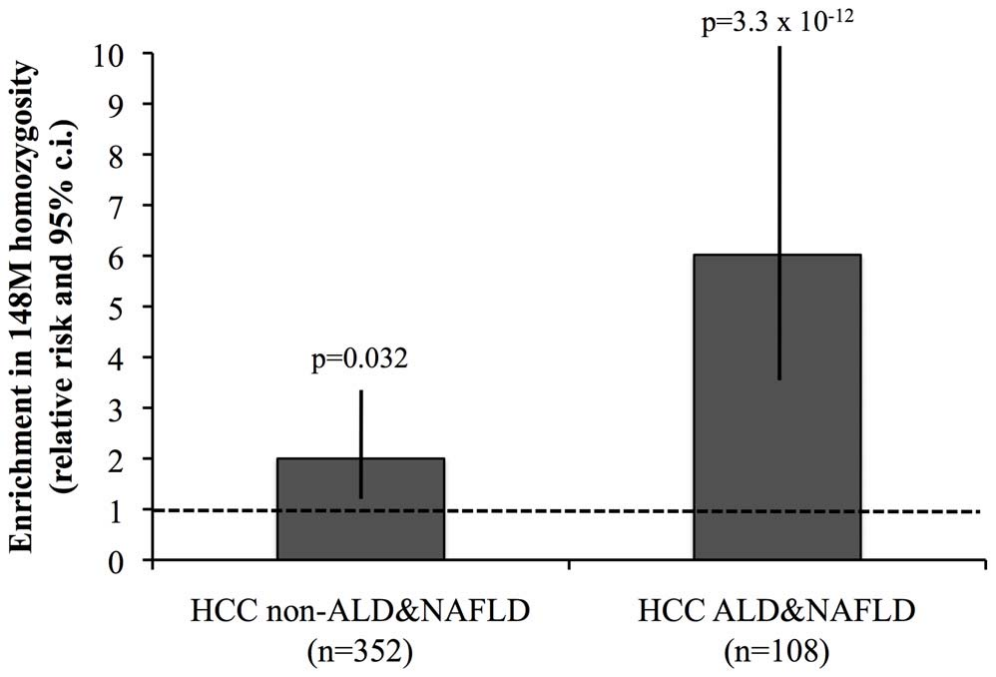
Enrichment in 148M homozygosity in patients with HCC vs. healthy controls. Relative risk and 95% confidence interval (c.i.) of 148M homozygosity in patients with HCC associated with liver diseases not directly related to steatohepatitis (non-ALD&NAFLD), and patients with HCC related to liver disease related to steatohepatitis (ALD&NAFLD).

The frequency distribution of the I148M was significantly different according to the etiology of liver disease (p = 3.3×10^−7^; p = 1.5×10^−6^ for 148M homozygosity). The prevalence of 148M homozygosity in HCC patients with ALD&NAFLD and other liver diseases and in healthy controls is shown in Figure S1. Homozygosity for the 148M PNPLA3 variant was significantly enriched in HCC patients, in particular in those with ALD&NAFLD, but also in those with other liver diseases (ALD&NAFLD: relative risk 5.9, 95% confidence interval (c.i.) 3.5–9.9; other liver diseases: relative risk 1.9, 95% c.i. 1.1–3.4; Figure 1). At logistic regression analysis, the association between 148M homozygosity and HCC was independent of age and gender (OR 3.5, 95% c.i. 1.8–7.2, p = 2.3×10^−4^), both in ALD&NAFLD (OR 10.6, 95% c.i. 4.8–25.3; p = 6.1×10^−10^), and in non ALD&NAFLD patients (OR 1.9, 95% c.i. 1.1–4.6; p = 0.05).

### I148M PNPLA3 polymorphism and clinical presentation of HCC

Due to the heterogeneity of disease etiologies influencing the clinical presentation, we next evaluated the effect of I148M on clinical presentation in patients subdivided according to the cause of liver disease (ALD&NAFLD vs. other etiologies; Table 2). In patients with ALD&NAFLD, PNPLA3 148M alleles were associated with younger age at diagnosis of HCC (p = 0.018; p = 0.040 under a recessive model), both in cirrhotic patients in follow-up and in incident cases (p = 0.036 and p = 0.044, respectively). These results are in line with a shorter time from the diagnosis of cirrhosis to HCC development in patients in follow-up (p = 0.05 for ALD&NAFLD, p = 0.030 under a recessive model). In addition, ALD&NAFLD patients carrying 148M alleles tended to develop HCC at a less advanced stage of liver disease (i.e. Child A cirrhosis: p = 0.035; p = ns at recessive model). The association remained significant in patients in ALD&NAFLD patients in regular follow-up (p = 0.042). Furthermore, the PNPLA3 148M allele was associated with a higher number of HCC lesions at presentation in ALD&NAFLD (p = 0.007; p = 0.049 in cases diagnosed in regular follow-up).

**Table 2:** Effect of I148M PNPLA3 variant on clinical presentation of HCC according to the etiology of liver disease (ALD & NAFLD vs. other etiologies).

	ALD & NAFLD				Other liver diseases			
PNPLA3 I148M	I/I	I/M	M/M	p value°	I/I	I/M	M/M	p value°
n =	22 (20)	47 (44)	39 (36)	-	172 (49)	140 (40)	40 (11)	-
Age years	67±7	64±9	62±6	0.018	67±10	65±10	67±9	0.18
Sex F	4 (18)	8 (17)	3 (8)	0.37	36 (21)	37 (26)	9 (22)	0.59
Time from cirrhosis years	2 {1–7}	2 {0–6}	2 {0–3}	0.05	5{1–10}	4 {1–11}	5{2–12}	0.26
Diabetes	10 (45)	16 (34)	17 (44)	0.55	45 (34)	22 (29)	8 (28)	0.42
Cirrhosis	20 (91)	44 (94)	36 (92)	0.92	164 (96)	139 (99)	39 (98)	0.17
Child B/C	12 (55)	11 (23)	12 (31)	0.035	29 (16)	39 (42)	7 (18)	0.86
Lesion number*	1 {1–2}	1 {1–2.8}	1 {1–3.5}	0.007	1{1–2}	1{1–2}	1{1–3}	0.98
Major lesion mm*	20 {20–44}	25 {18–45}	30 {20–62}	0.32	25{18–30}	25 {20–40}	28 {18–36}	0.56
Very earlyHCC*	8 (38)	12 (30)	10 (27)	0.39	45 (32)	24 (29)	9 (31)	0.89
AdvancedHCC**	14 (64)	19 (45)	27 (67)	0.07	71 (45)	70 (59)	14 (42)	0.36

(): % values, {}: median and interquartile range. HCC: hepatocellular carcinoma, ALD: alcoholic liver disease, NAFLD: nonalcoholic fatty liver disease, F: female, PNPLA3: patatin-like phosholipase domain-containing 3. °Additive model. * Available in 353 patients, ** available in 413 patients. Very early HCC and advanced / terminal HCC were defined according to the updated Barcelona Clinic Liver Cancer (BCLC) staging system and EASL/EORTC guidelines [1].

In contrast, PNPLA3 genotype did not influence clinical features at presentation in the overall series of HCC patients with other liver diseases (Table 2). However, when we stratified patients for the two major remaining etiologies (HBV and HCV, results are shown in Table S3), in patients with HBV we observed that PNPLA3 148M alleles were associated with younger age at presentation (p = 0.011; p = 0.032 under a recessive model), shorter time from cirrhosis to diagnosis (p = 0.045, p = 0.20 under a recessive model), and with presentation of HCC at a less advanced stage of liver disease (p = 0.032).

Histological diagnosis with the evaluation of HCC differentiation grade was available in 118 patients. In this subset of patients, PNPLA3 I148M was associated with higher Edmonson histological grade in the overall series (Edmonson grade II-IV: 17/22, 77% vs. 50/96, 52%; p = 0.03). There was a significant association between PNPLA3 148M homozygosity and higher HCC grade in patients with ALD&NAFLD (10/12, 83% vs. 10/22, 45%; p = 0.03), but not in those with other liver diseases (7/10, 70% vs. 40/74, 54%; p = 0.3). These data are in line with more aggressive histological and clinical presentation of HCC in ALD&NAFLD patients homozygous for PNPLA3 148M.

### I148M polymorphism and survival of HCC patients

**Figure 2:**
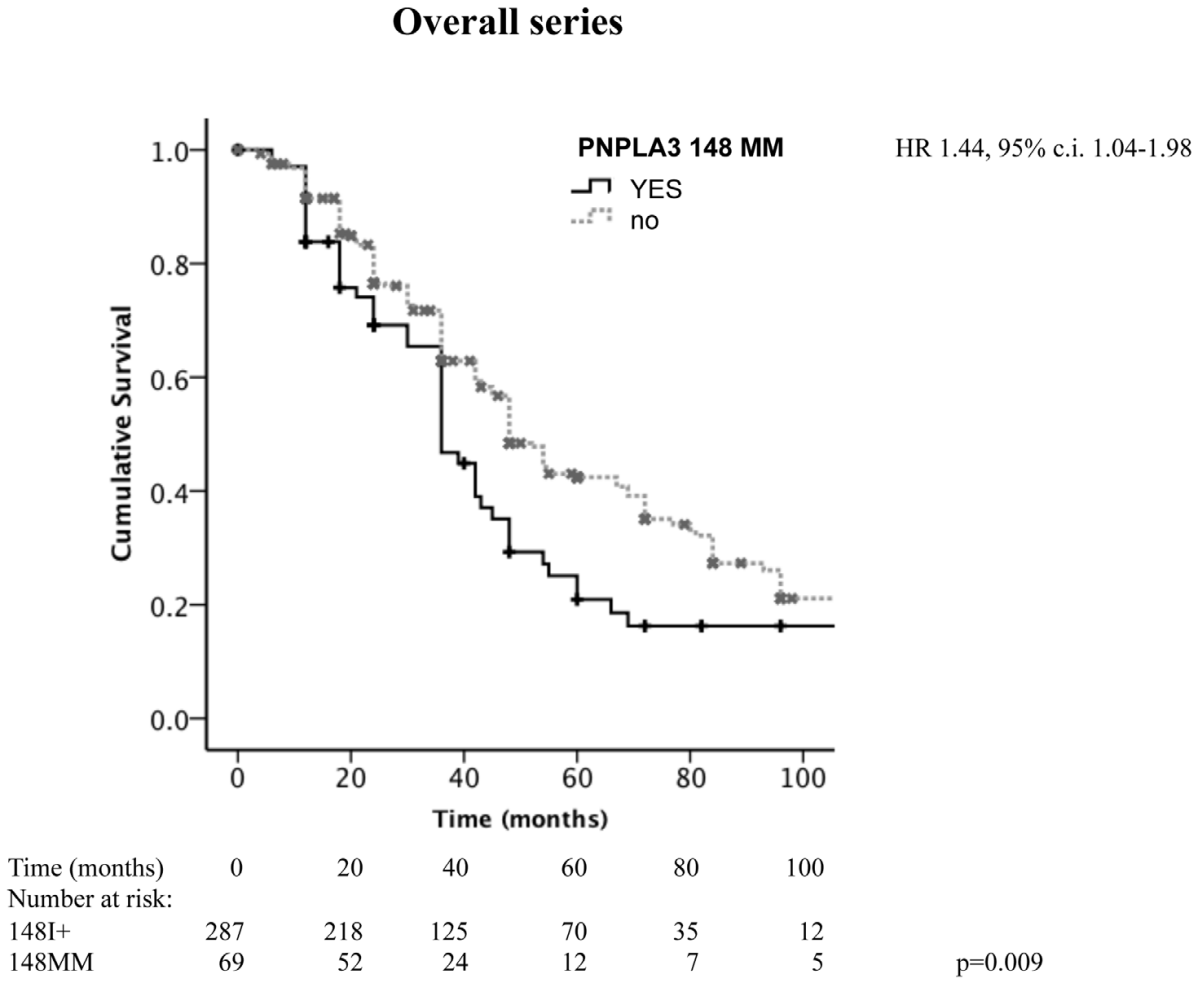
Kaplan-Meier estimates for survival in HCC patients subdivided according to the presence of homozygosity for PNPLA3 148M (p = 0.009 at Log-Rank test). Figures at the bottom refer to the number of patients still under observation at each time interval. HR: hazard ratio of death for homozygosity for the 148M allele, c.i.: confidence interval.

**Figure 3:**
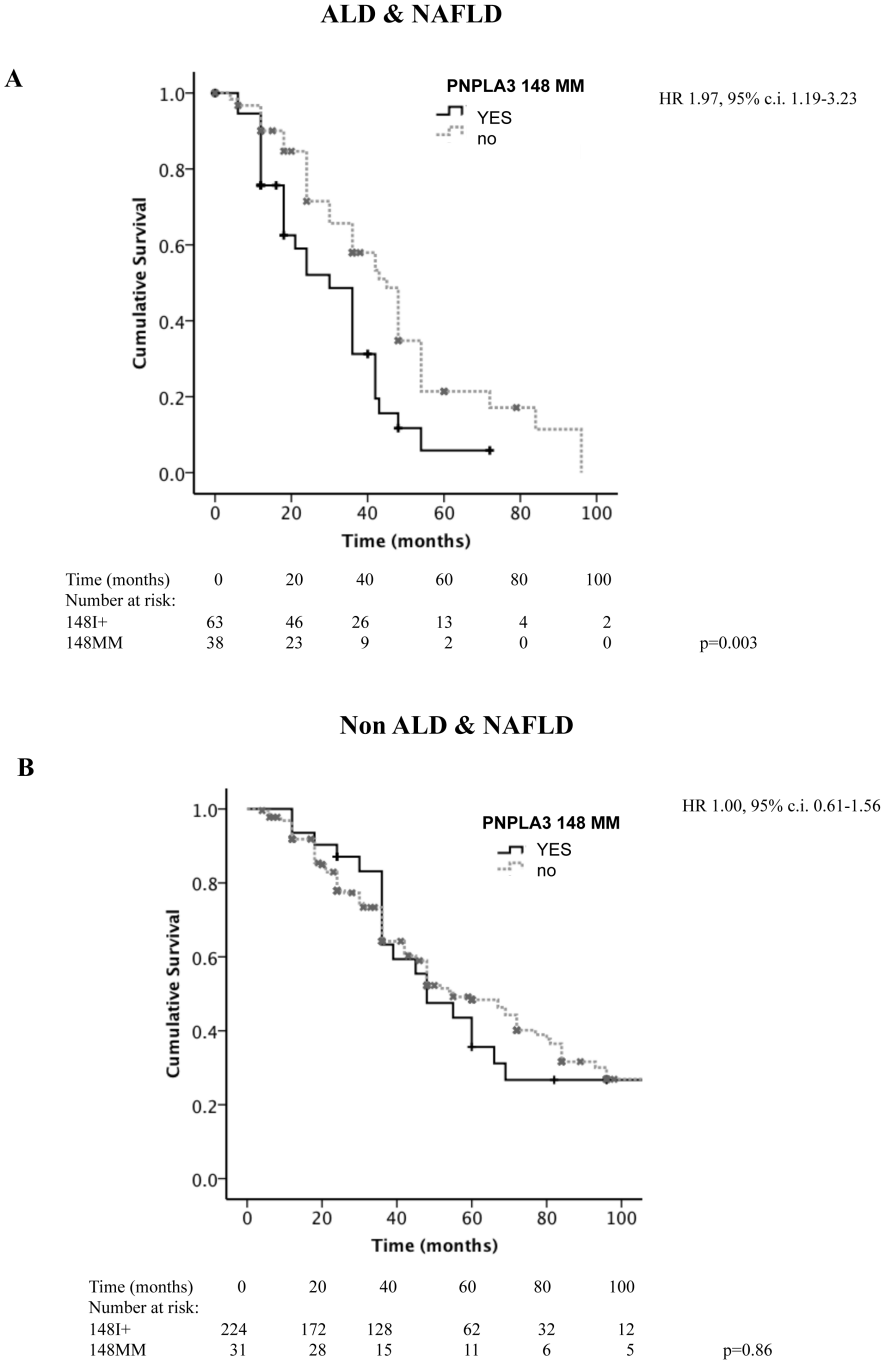
Kaplan-Meier estimates for survival in HCC patients subdivided according to the presence of homozygosity for PNPLA3 148M. Patients were stratified according to the etiology of liver disease: ALD&NAFLD (panel **A**; p = 0.003), and non-ALD&NAFLD (panel **B**; p = 0.86). Figures at the bottom refer to the number of patients still under observation at each time interval. HR: hazard ratio of death for homozygosity for the 148M allele, c.i.: confidence interval.

Considering the overall cohort, PNPLA3 148M homozygotes had a lower survival (p = 0.009 at Log-Rank test; median survival 36, 95% c.i. 30–42 vs. 48, 95% c.i. 43–52 months; Figure 2). ALD&NAFLD patients had a worse survival compared to other liver diseases (p = 2×10^−6^ at Log-Rank test; median survival 36, 95% c.i. 29–43 vs. 55, 95% c.i. 44–66 months). After stratification of patients according to the etiology of liver disease (ALD&NAFLD vs. others), homozygosity for PNPLA3 148M was associated with lower survival in ALD&NAFLD (p = 0.003 at Log-Rank test; median survival 30, 95% c.i. 20–39 vs. 45, 95% c.i. 38–52 months; Figure 3A), but not in patients with other etiologies (p = 0.86 at Log-Rank test; median survival 48, 95% c.i. 32–64 vs. 55, 95% c.i. 43–67 months; Figure 3B).

**Figure 4:**
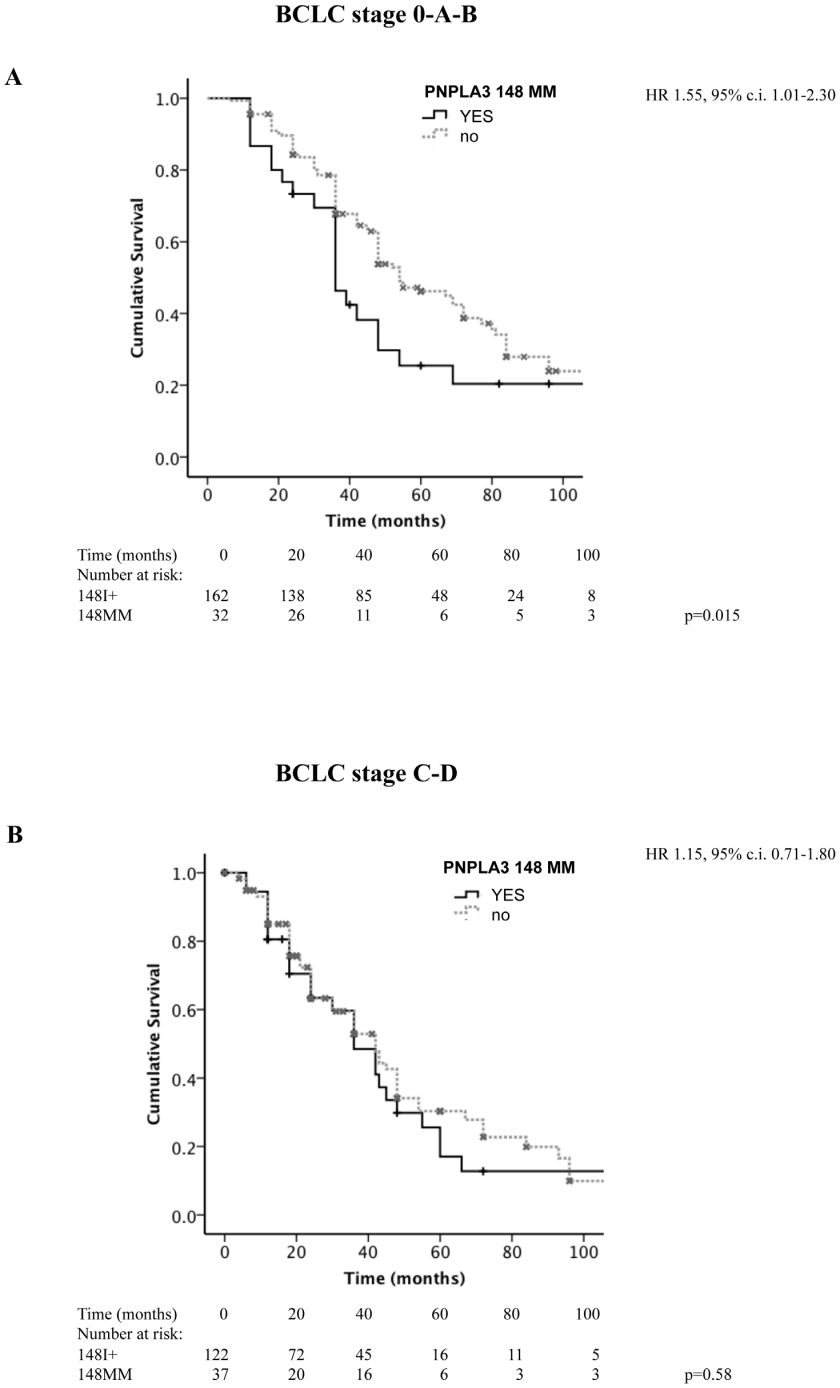
Kaplan-Meier estimates for survival in HCC patients subdivided according to the presence of homozygosity for PNPLA3 148M, stratified according to HCC stage at baseline. BCLC 0-A-B (panel **A**; p = 0.015), and BCLC stage C–D (panel **B**; p = 0.58). Figures at the bottom refer to the number of patients still under observation at each time interval. HR: hazard ratio of death for homozygosity for the 148M allele, c.i.: confidence interval.

Patients with advanced stage (BCLC stage C–D) had a worse survival compared to those presenting with less advanced, including intermediate stage (BCLC stage 0-A-B) disease (p = 9.2×10^−5^ at Log-Rank test; median survival 42, 95% c.i. 36–45 vs. 48, 95% c.i. 48–67 months). After stratification of patients according to the HCC stage at presentation (0-A-B vs. C–D), homozygosity for PNPLA3 148M was associated with lower survival in patients with less advanced (p = 0.015 at Log-Rank test; median survival 36, 95% c.i. 30–48 vs. 54, 95% c.i. 48–72 months; Figure 4A), but not in those with advanced disease (p = 0.57 at Log-Rank test; median survival 36, 95% c.i. 24–48 vs. 42, 95% c.i. 30–48 months; Figure 4B).

Predictors of death at multivariate Cox regression analysis in patients subdivided according to the etiology of liver disease (ALD&NAFLD vs. others) are shown in Table 3.

**Table 3:** Independent predictors of death at multivariate Cox regression analysis in 356 patients with HCC with available follow-up subdivided according to the etiology of underlying liver disease (ALD & NAFLD vs. others).

	ALD & NAFLD (n = 101)			Other liver diseases (n = 255)		
	HR	95% c.i.	p value	HR	95% c.i.	p value
Age years	1.00	0.97–1.03	0.94	1.02	1.00–1.04	0.05
Advanced stage	1.02	0.63–1.69	0.91	1.51	1.06–2.17	0.024
Diabetes	1.19	0.73–1.92	0.47	1.39	1.00–2.04	0.05
PNPLA3 148 MM	1.87	1.12–2.78	0.017	1.12	0.71–1.92	0.55

ALD: alcoholic liver disease, NAFLD: nonalcoholic fatty liver disease, Advanced stage: BCLC C-D, HR: hazard ratio of death, 95% c.i.: 95% confidence interval.

In ALD&NAFLD, homozygosity for the PNPLA3 148M allele was the only negative prognostic factor (hazard ratio (HR) 1.87, 95% c.i. 1.12–2.78), whereas in patients with other liver diseases, age, advanced stage, and type 2 diabetes negatively influenced survival.

## Discussion

In this paper, we confirm previous reports indicating that the PNPLA3 148M allele is a risk factor for HCC, in particular in patients with ALD&NAFLD. We also report novel data suggesting that HCCs arising in patients with ALD&NAFLD homozygous for the 148M PNPLA3 allele present earlier during the natural history of cirrhosis, are less differentiated, and have a larger diffusion at presentation, and a worse prognosis.

The study was prompted by accumulating evidence that PNPLA3 I148M influences both liver disease progression and carcinogenesis, representing a potentially useful biomarker for HCC risk stratification [3], [4], [5], [6], [7], [8], [9], [10], [31], whereas the clinical implications of carrying the I148M polymorphism for patients already diagnosed with HCC are still unknown.

Although the present study was not designed to evaluate the association of the I148M polymorphism on HCC risk, in line with previous results [3], [4], [5], [6], [7], [8], [9], [10], [31], the frequency of 148M was higher in HCC patients than in controls without liver disease. In particular, in ALD&NAFLD HCC patients, 148M homozygosity was observed in about one in three subjects, and it was enriched almost six-fold than in healthy control subjects. PNPLA3 148M predisposes to fibrosis progression [4], [18], [32], [33], but whether the effect on carcinogenesis is independent of cirrhosis is still debated [4], [6], [8], [25], [34]. On the other hand, data concordantly indicate that the PNPLA3 148M predisposes to HCC in ALD, and is a risk factor for HCC in obesity [3], [5], [6], [7], [8], [25]. The association of PNPLA3 148M with younger age, shorter history of cirrhosis, and less advanced liver disease at HCC diagnosis in ALD&NAFLD is therefore in line with direct carcinogenic activity of PNPLA3 148M, so that 148M homozygotes develop HCC before the other complications of cirrhosis.

In ALD&NAFLD patients, PNPLA3 148M homozygosity was associated with more diffuse HCC at diagnosis, as estimated by the number of detected lesions, with more advanced grade in subset of patients, and with reduced survival, representing the only independent prognostic factor in these patients. Overall mortality was chosen as the most appropriate outcome because is the most solid and undisputable clinical endpoint, and in addition the changes in treatment paradigm of HCC during the study period would have confounded the evaluation of the outcome of specific treatment approaches.

The effect of 148M homozygosity on survival was significant in the overall cohort, and in patients with ALD&NAFLD. Furthermore, it was also evident in patients who did not present with advanced or terminal HCC, suggesting that the 148M PNPLA3 allele may be associated with the development of more aggressive HCC, which are not cured or are not amenable to be cured by available therapies. Supporting our conclusions, after the submission of the present paper, it was independently reported that in another retrospective-prospective case series of Caucasians HCC patients from the US, who were mostly negative for HBV and HCV infection, homozygosity for the 148M allele was independently associated with reduced survival [35].

We cannot exclude that, especially in ALD where hepatic failure represents a common cause of death [8], the I148M polymorphism affected mortality by influencing the progression of liver disease rather than that of HCC. However, homozygosity for the 148M allele was more represented in younger patients with less advanced liver disease at baseline, rendering less likely that it was associated with excess mortality because of liver failure not secondary to HCC progression.

Due to the heterogeneity of patients evaluated, of treatment approaches, and the retrospective/prospective design, our results need to be evaluated in prospective cohorts of selected patients with early stages of HCC to minimize the effect of confounders, such as different treatment modalities, to assess the usefulness of the I148M polymorphism as a biomarker for stratification of HCC patients.

In conclusion, PNPLA3 148M is over-represented in HCC patients, in particular in those with ALD&NAFLD, where it is associated with earlier presentation at a less advanced stage of liver disease. Furthermore, in ALD&NAFLD, PNPLA3 148M is associated with more diffuse HCC at presentation, and with reduced survival. Additional studies are required to evaluate the mechanisms of hepatocarcinogenesis associated with PNPLA3 148M, and whether this genetic is associated with a specific biological subtype of HCC, and can predict the response to treatment.

## Supporting Information

Figure S1 **Prevalence of 148M homozygosity.** In the reference group of healthy subjects, patients with HCC associated with liver diseases not directly related to steatohepatitis (non-ALD&NAFLD), and patients with HCC related to liver disease related to steatohepatitis (ALD&NAFLD).
(TIF)

Table S1 **Demographic, clinical, and genetic features of subjects with complete evaluation of the neoplastic lesion at diagnosis, and with available follow-up, included in the study.**
(DOCX)

Table S2 **Demographic, clinical, and genetic features of HCC patients according to the etiology of liver disease.**
(DOCX)

Table S3 **Effect of I148M PNPLA3 variant on clinical presentation of HCC in patients with chronic HBV or chronic HBV infection.**
(DOCX)
