# Supplementary figures and images for: PNPLA3 I148M Polymorphism, Clinical Presentation, and Survival in Patients with Hepatocellular Carcinoma
Source: PLoS One. 2013 Oct 14;8(10):e75982. doi: 10.1371/journal.pone.0075982 (PMC3796509; doi:10.1371/journal.pone.0075982)

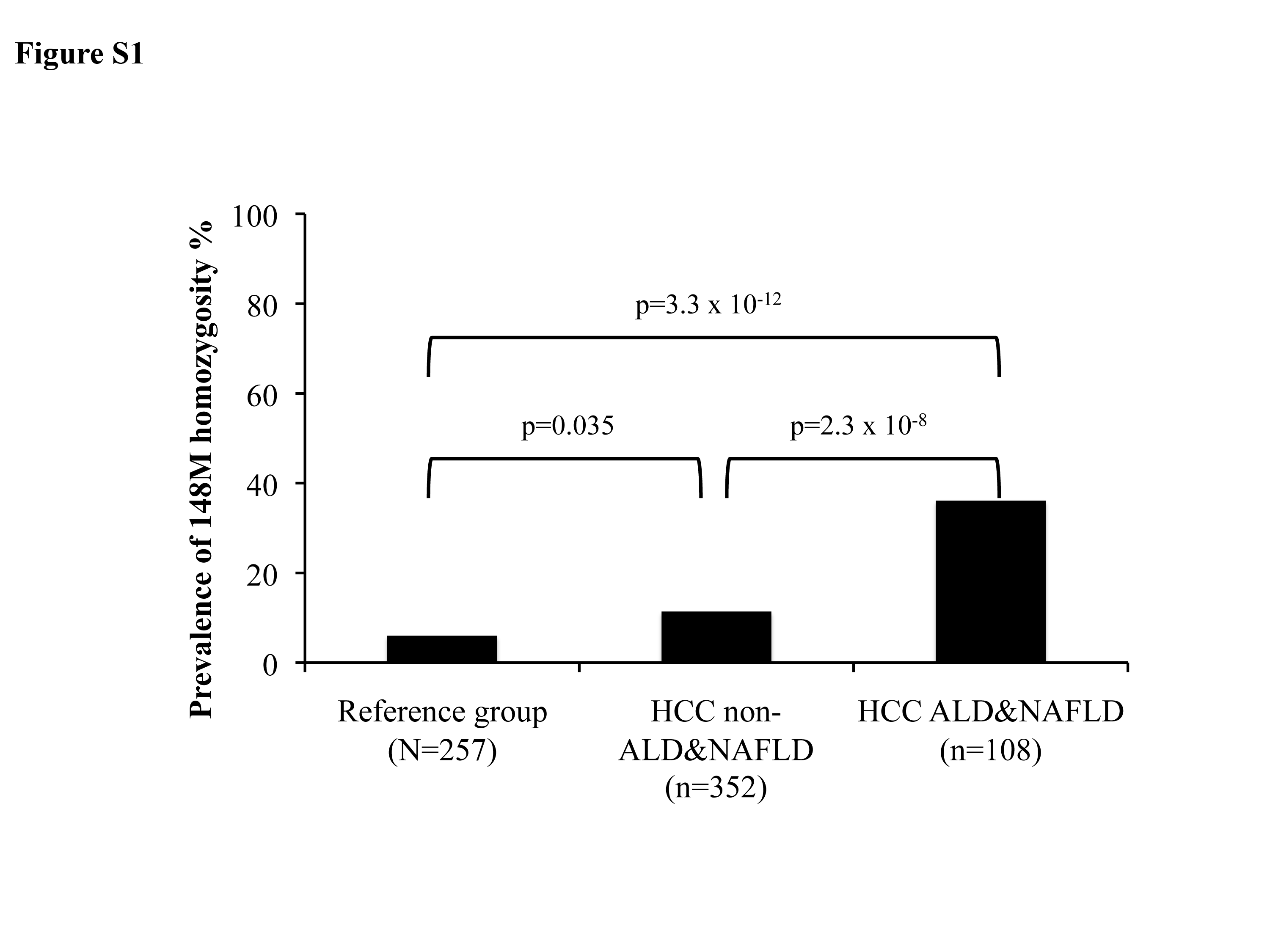

Supplement: Figure S1 — Prevalence of 148M homozygosity. In the reference group of healthy subjects, patients with HCC associated with liver diseases not directly related to steatohepatitis (non-ALD&NAFLD), and patients with HCC related to liver disease related to steatohepatitis (ALD&NAFLD). (TIF) [file pone.0075982.s001.tif]
