# Supplementary material for: PNPLA3 I148M Polymorphism, Clinical Presentation, and Survival in Patients with Hepatocellular Carcinoma
Source: PLoS One. 2013 Oct 14;8(10):e75982. doi: 10.1371/journal.pone.0075982 (PMC3796509; doi:10.1371/journal.pone.0075982)
Supplement: Table S1 — Demographic, clinical, and genetic features of subjects with complete evaluation of the neoplastic lesion at diagnosis, and with available follow-up, included in the study. (DOCX) [file pone.0075982.s002.docx]

**Table S1**. Demographic, clinical, and genetic features of subjects with complete evaluation of the neoplastic lesion at diagnosis, and with available follow-up, included in the study.

| n= | 353 |
| --- | --- |
| Age years | 66±9 |
| Sex F | 72 (20) |
| Diabetes | 116 (33) |
| HCV | 195 (55) |
| HBV | 48 (14) |
| Alcohol abuse | 76 (21) |
| Cirrhosis | 335 (95) |
| PNPLA3 I148M |  |
| I / I | 162 (46) |
| I / M | 125 (35) |
| M / M | 66 (19) |

(): % values; HCC: hepatocellular carcinoma; HCV: chronic hepatitis C virus infection, HBV: chronic hepatitis B virus infection (co-infected patients were considered in both categories), F: female, PNPLA3: patatin-like phosholipase domain-containing 3.
