# Supplementary material for: PNPLA3 I148M Polymorphism, Clinical Presentation, and Survival in Patients with Hepatocellular Carcinoma
Source: PLoS One. 2013 Oct 14;8(10):e75982. doi: 10.1371/journal.pone.0075982 (PMC3796509; doi:10.1371/journal.pone.0075982)
Supplement: Table S2 — Demographic, clinical, and genetic features of HCC patients according to the etiology of liver disease. (DOCX) [file pone.0075982.s003.docx]

**Table S2**. Demographic, clinical, and genetic features of HCC patients according to the etiology of liver disease.

| All patients (n=460) |  |  |  |  |  |  |  |  |
| --- | --- | --- | --- | --- | --- | --- | --- | --- |
| Etiology | HCV | HBV | C + B | ALD | NAFLD | HH | Crypto | p value |
| n= | 271 (59) | 57 (12) | 8 (2) | 80 (18) | 28 (6) | 10 (2) | 6 (1) | - |
| Age years | 68±9 | 59±10 | 61±9 | 64±7 | 65±10 | 72±8 | 62±15 | 2x10^-10^ |
| Sex F | 65 (24) | 10 (18) | 2 (25) | 5 (6) | 10 (36) | 2 (20) | 3 (50) | 0.002 |
| Cirrhosis | 262 (97) | 56 (98) | 8 (100) | 76 (95) | 24 (86) | 10 (100) | 6 (100) | 0.09 |
| Child B/C | 63 (23) | 11 (19) | 5 (63) | 23 (29) | 2 (7) | 0 | 3 (50) | 0.004 |
| Patients with complete data (n=353) |  |  |  |  |  |  |  |  |
| n= | 189 | 46 | 2 | 78 | 26 | 10 | 4 | - |
| Lesions number* | 1 {1-2} | 1 {1-3.5} | 1 {1-4} | 1 {1-3} | 1 {1-3} | 1 {1-1} | 2 {1-4.5} | 0.22 |
| Major lesion mm* | 25 {19-33} | 25 {19-33} | 26 {22-30} | 30 {20-50} | 24 {18-40} | 22 {15-60} | 26 {15-61} | 0.040 |
| Very early  (stage 0) HCC* | 61 (32) | 13 (29) | 0 | 24 (32) | 6 (27) | 4 (40) | 0 | 0.48 |
| Advanced / terminal  (stage C/D) HCC** | 116 (50) | 23 (44) | 6 (86) | 50 (63) | 10 (42) | 5 (50) | 5 (100) | 0.016 |
| Follow-up months* | 36 {24-60} | 41{18-55} | 60 {12-98} | 30 {18-48} | 24 {12-42} | 36 {15-66} | 21 {6-33} | 0.007 |
| PNPLA3 I148M (n=460) |  |  |  |  |  |  |  | 3.3x10^-7^ |
| I / I | 132 (48) | 31 (54) | 2 (25) | 18 (22) | 4 (14) | 4 (40) | 3 (50) |  |
| I / M | 107 (40) | 19 (34) | 6 (75) | 32 (40) | 15 (54) | 5 (50) | 3 (50) |  |
| M / M | 32 (12) | 7 (12) | 0 | 30 (38) | 9 (32) | 1 (10) | 0 |  |

(): % values, {}: median and interquartile range. HCC: hepatocellular carcinoma, HCV: hepatitis C virus, HBV: hepatitis B virus, C+ B: HCV + HBV, ALD: alcoholic liver disease, NAFLD: nonalcoholic fatty liver disease, HH: hereditary hemochromatosis, Crypto: cryptogenic, F: female, PNPLA3: patatin-like phosholipase domain-containing 3. * Available in 353 patients, ** available in 413 patients (some patients could be classified as terminal stage because of the presence of Child B/C cirrhosis even if characterization of HCC at diagnosis was not available). Very early HCC and advanced / terminal HCC were defined according to the updated Barcelona Clinic Liver Cancer (BCLC) staging system and EASL/EORTC guidelines [1].
