# Supplementary material for: PNPLA3 I148M Polymorphism, Clinical Presentation, and Survival in Patients with Hepatocellular Carcinoma
Source: PLoS One. 2013 Oct 14;8(10):e75982. doi: 10.1371/journal.pone.0075982 (PMC3796509; doi:10.1371/journal.pone.0075982)
Supplement: Table S3 — Effect of I148M PNPLA3 variant on clinical presentation of HCC in patients with chronic HBV or chronic HBV infection. (DOCX) [file pone.0075982.s004.docx]

**Table S3**. Effect of I148M PNPLA3 variant on clinical presentation of HCC in patients with chronic HBV or chronic HBV infection.

|  | HCV |  | |  |  | HBV |  |  | |  |
| --- | --- | --- | --- | --- | --- | --- | --- | --- | --- | --- |
| PNPLA3 I148M | I / I | | I / M | M / M | p value° | I / I | I / M | | M / M | p value° |
| n= | 132 (49) | | 107 (39) | 32 (12) | - | 31 (54) | 19 (33) | | 7 (12) | - |
| Age years | 68±9 | | 68±9 | 69±9 | 0.91 | 63±8 | 56±11 | | 55±6 | 0.011 |
| Sex F | 30 (23) | | 28 (26) | 7 (22) | 0.79 | 4 (13) | 4 (21) | | 2 (29) | 0.27 |
| Time from cirrhosis years | 5 {1-9} | | 4 {1-9} | 5 {4-10} | 0.29 | 5 {1-10} | 1.5 {0-5} | | 2 {0-4} | 0.045 |
| Diabetes | 32 (24) | | 17 (16) | 5 (16) | 0.22 | 9 (29) | 2 (11) | | 2 (29) | 0.30 |
| Cirrhosis | 125 (95) | | 106 (97) | 31 (99) | 0.26 | 30 (97) | 19 (100) | | 7 (100) | 0.65 |
| Child B/C | 42 (32) | | 41 (38) | 6 (19) | 0.09 | 5 (16) | 8 (42) | | 0 | 0.032 |
| Lesion number* | 1 {1-2} | | 1 {1-2} | 1 {1-2} | 0.97 | 1{1-3} | 2 {1-4} | | 1 {1-5} | 0.73 |
| Major lesion mm* | 25 {19-30} | | 25 {19-36} | 29 {18-36} | 0.61 | 25 {16-30} | 25 {20-47} | | 20 {20-40} | 0.94 |
| Very early  HCC* | 35 (33) | | 20 (32) | 6 (28) | 0.90 | 9 (33) | 2 (18) | | 2 (29) | 0.70 |
| Advanced  HCC** | 55 (45) | | 51 (58) | 10 (40) | 0.07 | 10 (32) | 10 (59) | | 3 (43) | 0.21 |

(): % values, {}: median and interquartile range. HCC: hepatocellular carcinoma, HCV: hepatitis C virus, HBV: hepatitis B virus, ALD: alcoholic liver disease, NAFLD: nonalcoholic fatty liver disease, F: female, PNPLA3: patatin-like phosholipase domain-containing 3. °Additive model. * Available in 353 patients, ** available in 413 patients. Very early HCC and advanced / terminal HCC were defined according to the updated Barcelona Clinic Liver Cancer (BCLC) staging system and EASL/EORTC guidelines [1]. Very early and early stage HCC (BCLC stage 0 and A) was treated by surgery, radiofrequency ablation, or percutaneous ethanol injection (during the first years of the study); liver transplantation was considered in at risk patients without exclusion criteria and within the Milano criteria; intermediate stage HCC (BCLC stage B) was treated by transarterial chemoembolization (TACE) alone or in combination with percutaneous ablative procedures; advanced HCC was treated by supportive care (or palliative TACE in very selected cases) and sorafenib when it became available (n=31 patients included); terminal stage HCC (BCLC stage D) was treated by supportive care.
